# Genetic Diversity and Selection Signatures in Jianchang Black Goats Revealed by Whole-Genome Sequencing Data

**DOI:** 10.3390/ani12182365

**Published:** 2022-09-10

**Authors:** Xueliang Sun, Jiazhong Guo, Li Li, Tao Zhong, Linjie Wang, Siyuan Zhan, Juan Lu, Decheng Wang, Dinghui Dai, George E. Liu, Hongping Zhang

**Affiliations:** 1 Key Laboratory of Livestock and Poultry Multi-Omics, Ministry of Agriculture and Rural Affairs, College of Animal Science and Technology, Sichuan Agricultural University, Chengdu 611130, China; 2 Farm Animal Genetic Resources Exploration and Innovation Key Laboratory of Sichuan Province, Sichuan Agricultural University, Chengdu 611130, China; 3 Bureau of Agriculture and Rural Affairs of Huidong County, Huidong 615200, China; 4 Animal Genomics and Improvement Laboratory, BARC, Agricultural Research Service, USDA, Beltsville, MD 20705, USA

**Keywords:** Chinese black goats, whole-genome sequencing, genetic diversity, ROH, genomic inbreeding coefficients, selection signature

## Abstract

**Simple Summary:**

Jianchang Black goats are one of the indigenous black goat breeds in China. They are known for their crude feed tolerance but have a low growth rate and small body size. However, little is known about the genetic composition of Jianchang Black goats. The purpose of this study was to systematically examine the genetic composition of this breed by conducting multiple population genetic analyses. Our results revealed that Jintang Black and Yunshang Black goats showed a close genetic relationship with a non-negligible amount of gene flows but were genetically distant from Jianchang Black goats. Jianchang Black goats generally showed moderate inbreeding levels. Importantly, we identified several putatively selected genes that may be potentially related to immunity, behavior, and reproduction in this breed. The current study provides valuable references and genomic resources for the future breeding of Jianchang Black goats.

**Abstract:**

Understanding the genetic composition of indigenous goats is essential to promote the scientific conservation and sustainable utilization of these breeds. The Jianchang Black (JC) goat, a Chinese native breed, is solid black and exhibits crude feed tolerance, but is characterized by a low growth rate and small body size. Based on the whole-genome sequencing data for 30 JC, 41 Jintang Black (JT), and 40 Yunshang Black (YS) goats, and 21 Bezoar ibexes, here, we investigated the genetic composition of JC goats by conducting analyses of the population structure, runs of homozygosity (ROH), genomic inbreeding, and selection signature. Our results revealed that JT and YS showed a close genetic relationship with a non-negligible amount of gene flows but were genetically distant from JC, apart from Bezoars. An average of 2039 ROHs were present in the autosomal genome per individual. The ROH-based inbreeding estimates in JC goats generally showed moderate values ranging from 0.134 to 0.264, mainly due to rapid declines in the effective population size during recent generations. The annotated genes (e.g., *IL2*, *IL7*, and *KIT*) overlapping with ROH islands were significantly enriched in immune-related biological processes. Further, we found 61 genes (e.g., *STIM1*, *MYO9A*, and *KHDRBS2*) under positive selection in JC goats via three complementary approaches, which may underly genetic adaptations to local environmental conditions. Our findings provided references for the conservation and sustainable utilization of JC goats.

## 1. Introduction

As a common livestock species, domestic goats (*Capra hircus*) are widely distributed across five continents, especially in Asia and Africa, which has resulted in an extensive phenotypic diversity among different breeds [1,2]. Although modern goats are mainly bred for meat, milk, fiber, and leather production, some appearance traits (e.g., coat color [3]) associated with socio-cultural preference are also important and contribute to the economic value of products in goats. For example, black goats, such as Jianchang Black goats (JC), Jintang Black goats (JT, also called Chuanzhong Black goats), and Yunshang Black goats (YS), are very popular in Southwest China, partly due to the coat color preference by farmers. Furthermore, it is relatively simple to breed black goats by phenotypic selection because the black coat color is usually recessive compared with other coat colors (e.g., white and yellow/red) in goats. Another important reason is that black goats (e.g., YS) often have tender meat with a unique flavor [4].

China is now the largest goat producer globally [1] and has a number of indigenous breeds raised in diverse environmental conditions with different precipitations, temperatures, oxygen contents, and ultraviolet intensities. For example, the JC goat mentioned above is a Chinese indigenous breed, originating from Huidong County and Huili City in the Liangshan Yi Autonomous Prefecture. Besides the solid black coat color, this breed has the characteristics of crude feed tolerance but a low growth rate and small body size. However, little is known about the genetic diversity of this breed. More importantly, its population size is declining mainly due to a lack of high commercial value and the loss of smallholder farmers. To our knowledge, the JT goat (native to Jintang County and neighboring regions on the Chengdu Plain), which is known for its large body size, has been introduced to Huidong County to genetically improve the body size and growth rate of JC goats in recent years. Furthermore, the YS goat, a meat-producing breed that originated from crossing Chinese indigenous Yunling goats and Nubian goats [5], is also intriguing because of the desirable characteristics mentioned above [4]. Although historical records briefly describe that these breeds originated from different geographical locations, the genetic relationships between these breeds are not well understood.

A genome-wide assessment of the genetic diversity and population structure is crucial for the conservation and utilization of the genetic resources of livestock species. Apart from classical metrics, runs of homozygosity (ROH) have been widely applied to characterize inbreeding levels and selection signals in livestock genetics over recent years. ROH refers to consecutive stretches of homozygous genotypes in the genome, resulting from the inheritance of the same ancestral haplotype from both parents [6]. Although other evolutionary factors (e.g., selection and linkage disequilibrium) also contribute to the ROH formation at the population level, inbreeding is a primary force driving the occurrence of ROHs. Thus, the proportion of the total genome encompassing ROHs can be an index to measure the actual inbreeding levels of individuals and populations [7]. It is also widely accepted that short ROHs usually result from ancient inbreeding, whereas long ROHs generally result from recent inbreeding [8]. Accordingly, ROHs with different lengths can reflect historical inbreeding events at different generations, which is an advantage of ROH-based estimates of inbreeding compared with other molecular inbreeding estimates, mainly including those derived from genomic kinship matrixes [9]. Therefore, ROH-based inbreeding coefficients are now widely used in a variety of animal species, such as pigs [10], cattle [11], sheep [12], and goats [13,14]. Furthermore, ROH islands (i.e., ROHs are common between individuals) at the population level can be an indicator of the identification of signatures of selection [10,12,14,15], demonstrating that ROH is a versatile statistic. However, genome-wide patterns of ROHs and ROH islands in JC goats remain unknown.

Here, we generated whole-genome sequencing (WGS) data for 30 JC goats from a core breeding farm in Huidong County. Together with the published data from 41 JT, 40 YS, and 21 Bezoar ibexes, the main aim of this study was to systematically investigate the genetic compositions of JC goats by conducting analyses of the population structure, genetic diversity, ROH, genomic inbreeding, and selection signature.

## 2. Materials and Methods

### 2.1. Sample Collection and Whole-Genome Sequencing Data

The blood samples of 30 male goats were collected from a core Jianchang Black (JC) goat breeding farm at Huidong County, the Liangshan Yi Autonomous Prefecture, China. We released the animals after sampling. Genomic DNA was extracted from whole blood samples using the E.Z.N.A.^®^ Blood DNA Kit (OMEGA BIO-TEK, Norcross, GA, USA). The paired-end libraries with an average insert size of 500 bp were constructed for each animal, and whole-genome sequencing was performed using Illumina NovaSeq 6000 instruments at Novogene (Beijing, China).

We then examined the genetic relationship between JC goats and other black breeds by using our previously generated WGS data for 41 JT goats [16,17] (NCBI accession numbers: PRJNA548681 and PRJNA734084). We also downloaded WGS data for 40 YS goats [5] (NCBI accession number: PRJNA611688) and 21 Bezoar ibexes [18] (NCBI accession number: PRJEB3136).

### 2.2. Alignment of Short-Reads and Variant Calling

For the alignment of short reads and short variant calling, we applied the bioinformatics pipelines described in our previous study [16]. Briefly, high-quality reads were aligned against the goat reference genome (the ARS1 assembly [19]) using BWA [20], followed by the removal of duplicated reads and local realignment around existing Indels and base quality score recalibration using Genome Analysis Toolkit (GATK, v 4.0.5.2) [21]. We first applied the GATK HaplotypeCaller module to detect short variants (i.e., SNPs and Indels) and merged them using CombineGVCFs. The high-quality short variants were detected by conducting joint calling of all gVCF via the GenotypeGVCFs module followed by VariantFiltration (the criteria: QUAL < 100.0, QD < 2.0, MQ < 40.0, FS > 60.0, SOR > 3.0, MQRankSum < −12.5, and ReadPosRankSum < −8.0) in GATK. The final variant dataset was obtained after discarding the variants with a minor allele frequency (MAF) < 0.05 and missing genotype > 10% using VCFtools (v0.1.16) [22]. The SnpEff [23] software (v4.3) was used for SNP variant annotation and effect prediction.

### 2.3. Phylogenetic and Population Structure Analysis

The genome-wide fixation index (i.e., Weir and Cockerham’s *F*_ST_ estimator [24]) in 10-kb non-overlapping windows was calculated using VCFtools [22]. Before conducting population structure and genetic relationship analyses, the total biallelic SNPs were pruned based on linkage disequilibrium (LD) using the ‘--indep-pairwise 50 10 0.2’ command in PLINK1.9 [25]. We constructed a neighbor-joining phylogenetic tree with MEGA X [26] after calculating the identity-by-state matrix between all 132 goat samples using PLINK1.9. The phylogenetic tree was then visualized using FigTree (http://tree.bio.ed.ac.uk/software/figtree/, accessed on 2 June 2021, v1.4.4). We also conducted principal component analysis (PCA) to analyze the genetic relationships via the smartpca program in EIGENSOFT (v6.1) [27]. Population structure analysis and individual clustering were carried out using ADMIXTURE (v1.3) [28] for K values from 2 to 4.

### 2.4. Genetic Diversity, Linkage Disequilibrium, and Genome-Wide Detection of ROHs

We used VCFtools to calculate the genome-wide nucleotide diversity (π) in 10-kb non-overlapping windows in JC goats. The genome-wide observed homozygosity (H_o_) and expected homozygosity (H_e_) at each SNP site were calculated using the –hardy option in PLINK1.9. We employed PopLDdecay (v3.41) with the command “−MaxDist 200 −MAF 0.05” [29] to examine linkage disequilibrium (LD) decay between SNPs in a population. To obtain genomic relatedness among JC goats, GCTA (v1.92) [30] with the command ‘--make-grm’ was used to calculate a genomic relationship matrix (GRM).

According to the guidelines from a recent work [31], we did not apply SNP pruning before ROH detection. The PLINK1.9 with the --homozyg command was used for the detection of ROHs in JC goats and other populations, respectively. The parameters were: ‘--homozyg-snp 10 --homozyg-kb 100 --homozyg-density 10 --homozyg-gap 100 --homozyg-window-snp 50 --homozyg-window-het 1 --homozyg-window-missing 5 --homozyg-window-threshold 0.05’, as described in the previous studies using WGS data [14,32]. Because ROHs with different lengths provide information on inbreeding at different past generations [33], the identified ROHs were divided into four length categories: 0.1–0.2 Mb, 0.2–0.5 Mb, 0.5–1 Mb, and > 1 Mb.

### 2.5. Estimation of Effective Population Size and Genomic Inbreeding Coefficients

The effective population size (N_e_) of JC goats in recent generations was estimated using SNeP (v1.1) [34] with default parameters. We calculated the ROH-based inbreeding coefficient (F_ROH_) in JC goats, defined as the fraction of the autosomal genome (a total length of 2,466,191,353 bp in the ARS1 assembly) covered by the total ROH in each animal genome. Based on the four ROH length categories defined above, we further calculated F_ROH 0.1–0.2 Mb_, F_ROH 0.2–0.5 Mb_, F_ROH 0.5–1 Mb_, and F_ROH > 1 Mb_, which corresponds to 250–500 generations, 100–250 generations, 50–100 generations, and 50 generations ago, respectively. We used PLINK1.9 to calculate the excess of homozygosity inbreeding coefficient (F_HOM_), which is also commonly used in livestock genetics.

### 2.6. Genome-Wide Identification of ROH Islands and Selection Signals

Here, the ROH segments shared by at least 50% of the samples were regarded as an indication of possible ROH islands (the number of SNPs ≥ 10 and the length ≥ 100 bp) throughout the genome. The R package detectRUNS (v0.9.6) [35] was applied to detect ROH islands shared among individuals based on the raw results obtained using PLINK1.9. We used the R package clusterProfiler (v4.4.1) [36] to perform the functional enrichment analyses of the genes overlapping with the putative ROH islands. The GO terms with a *p* value < 0.05 were considered significantly enriched.

Here, we used three complementary population statistics (i.e., *F*_ST_, ROH islands, and iHH12 [37]) to detect genomic regions under selection. As described in our previous work [16], the selscan software (v2.0.0) [38] was used to calculate the haplotype homozygosity-based statistic iHH12 with 10-kb non-overlapping windows. We first retained the outlier windows showing extremely high *F*_ST_ or iHH12 values (corresponding to the 0.5% right end of the tail). After genome annotations, the genes overlapping with the outlier regions shared by three statistics were finally regarded as the plausible selected genes. As mentioned above, we performed the functional enrichment analyses of the selected genes using *clusterProfiler* [36].

## 3. Results

### 3.1. Abundant Genomic Variants in Three Black Goat Breeds and Bezoars

The alignment of the short-read WGS data for 111 animals (i.e., 30 JC, 41 JT, and 40 YS goats) from three black goat populations and 21 Bezoars yielded an average sequencing depth of 10.47× (4.28–20.49×) per individual (Supplementary Materials Table S1). At the meta-population level, a total of 16,469,520 SNPs (16,376,056 biallelic and 93,464 multiallelic) and 1,476,196 indels were detected across the autosomal genome. The functional annotation analysis showed that the biallelic SNPs were majorly located in intergenic regions (45.67%) or intron regions (43.98%). By contrast, the biallelic SNPs in exon regions only accounted for 0.89% (Supplementary Materials Table S2). Moreover, a total of 12,163,553 biallelic SNPs were present in the autosomal genomes of 30 JC goats.

### 3.2. JC Was Genetically Distinct from Two Other Black Breeds in Southwest China

**Figure 1:**
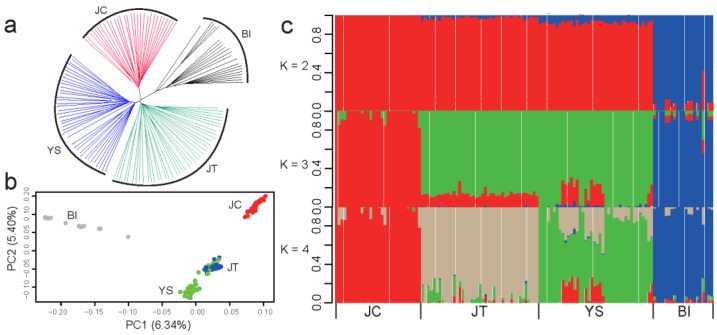
Genetic relationships and population structure of three black Chinese goat breeds and Bezoar ibexes. (**a**) Neighbor-joining (NJ) phylogenetic tree of three black Chinese goat breeds (i.e., Jianchang Black [JC], Jintang Black [JT], and Yunshang Black [YS]) and 21 Bezoars (BI) based on the identical-by-state (IBS) distance. (**b**) Principal component analysis (the first and second principal components [PC1 and PC2]) of 111 domestic goats and 21 Bezoars. (**c**) Proportions of genetic ancestry for 111 domestic goats and 21 Bezoars with K = 2~4 (K represents the number of inferred ancestral populations).

A neighbor-joining (NJ) phylogenetic tree using 1,386,488 independent biallelic SNPs (LD, *r^2^* > 0.2) showed that JT and YS breeds grouped closely, although three black goat breeds and Bezoars all formed their individual clusters (Figure 1a). The first principal component (PC), explaining 6.34% of the total genetic variations, distinguished three black goat breeds and Bezoars. The second PC, accounting for 5.40% of the total genetic variation, separated JC from JT and YS populations (Figure 1b). Moreover, several individuals from YS mixed with the JT breed, indicating gene flows between YS and JT breeds. The admixture analysis revealed K = 4 (cross-validation error = 0.557, Supplementary Materials Figure S1) as the most likely number of genetically distinct populations for 132 samples (Figure 1c). It also confirmed that JC has a unique pattern of genomic variations, and that a non-negligible amount of gene flows occurred between YS and JT breeds. Via the genome-wide *F*_ST_ in 10-kb sliding windows based on a total of 16,376,056 biallelic SNPs (Supplementary Materials Figure S2), we observed strong genetic differentiation between JC and the Bezoar ibex (average weighted *F*_ST_ = 0.216), which was significantly higher than between JC and YS (average weighted *F*_ST_ = 0.120) and JT (average weighted *F*_ST_ = 0.107) (Kruskal–Wallis rank sum test, *p* < 2.2 × 10^−16^). In contrast, there was a mild divergence between JT and YS (average weighted *F*_ST_ = 0.051).

### 3.3. Genetic Diversity Metrics and the Genome-Wide Pattern of ROH in JC Goats

Given that the WGS data for 30 JC goats were generated in this study, we focused on investigating the genetic diversity in JC goats. Most of the JC goats were not closely related based on a GRM (Supplementary Materials Table S3). The total average observed (H_o_) and expected (H_e_) heterozygosity were 0.233 and 0.308 across all the biallelic SNP sites. The genome-wide mean and median π values in a 10 kb sliding window were 1.47 × 10^−3^ and 1.18 × 10^−3^, respectively, in non-overlapping windows of 10 kb across the autosomes. The LD analysis revealed that the genome-wide mean r^2^ decreased rapidly from 0.57 between SNP pairs at a distance of 10 bp to 0.06 at 100 kb (Supplementary Materials Figure S3).

**Figure 2:**
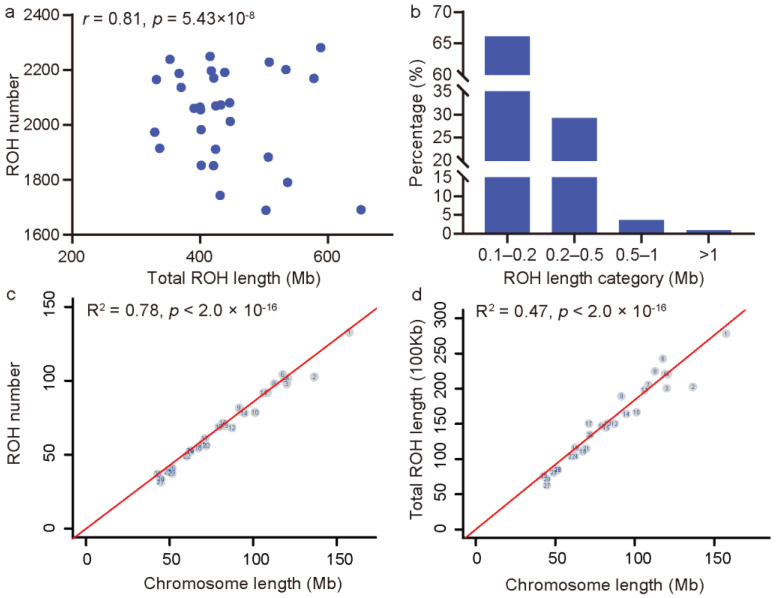
Summary of ROH detected in JC goats. (**a**) The summary of the total ROH number and length in the genome of each JC goat. (**b**) Proportions of the ROHs with different length classes. (**c**) and (**d**) The linear relationship between the number of ROH on each chromosome and the chromosome length. The numbers in the circles are the chromosome numbers. The regression slopes were 0.857 for the ROH number and 1.841 for the total ROH length on each chromosome.

We detected a total of 61,184 ROHs in all 30 JC goats, and the average number of ROHs per animal was 2039, with a range of 1693 to 2289 (Figure 2a). The total ROH length per individual varied from 329.06 Mb to 652.04 Mb (mean = 440.16 Mb), which was positively correlated with the total ROH number in each animal (Pearson’s *r* = 0.81, *p* = 5.43 × 10^−8^) (Figure 2a). Furthermore, the analysis of variance showed that the total ROH number and total ROH length per individual in JC goats were both significantly (*p* < 2.20 × 10^−16^ and *p* = 2.92 × 10^−8^) higher than those in JT (average total number = 1167 and average total length = 300.89 Mb), YS (average total number = 1059 and average total length = 300.08 Mb), and Bezoars (average number = 1195 and average total length = 444.28 Mb) (Supplementary Materials Table S4).

The predefined minimum and overall mean length of ROHs in JC goats were 100 kb and 215.82 kb, respectively, and the longest ROH was 8.31 Mb in size (37,288 SNPs) on Chromosome 9. Considering the length classes, the ROHs ≤ 0.2 Mb in size accounted for 66.07%, whereas the ROHs ≥ 1 Mb accounted for only 0.98% (Figure 2b). Briefly, the length of all the ROHs approximated an L-shaped distribution in JC goats. At the chromosome level, Chromosome 1 showed the highest number of ROHs (average value = 133) and total ROH length (average value = 27.82 Mb) (Figure 2c,d). A linear regression analysis revealed that the number of ROHs on the chromosome significantly increased with the chromosome length (*p* < 2.0 × 10^−16^, F-test, R^2^ = 0.78) (Figure 2c), which was also true for the total length of ROHs on each chromosome (*p* < 2.0 × 10^−16^, F-test, R^2^ = 0.47) (Figure 2d). The significantly positive linear relationships between the chromosome length and the total ROH number or the total ROH length per chromosome were also observed in JT, YS, and Bezoars (Supplementary Materials Table S5).

### 3.4. Effective Population Size and Genomic-Based Inbreeding Coefficients in JC Goats

**Figure 3:**
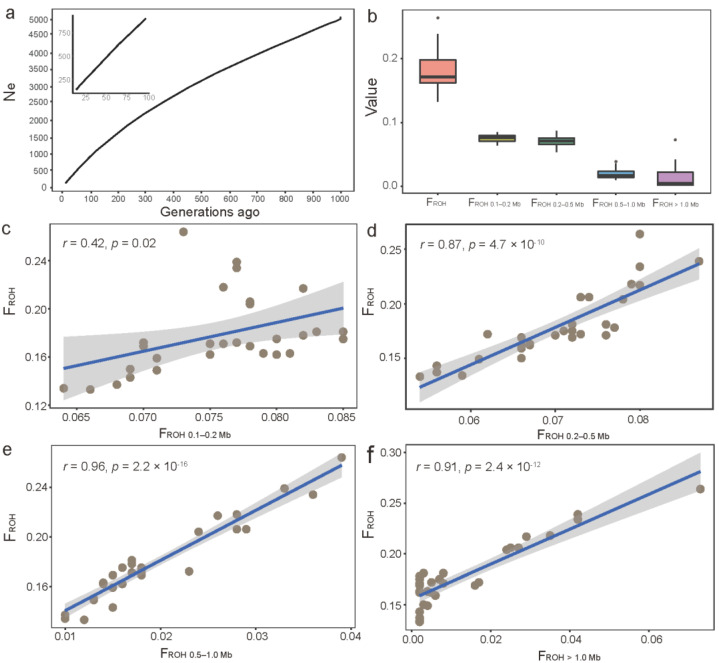
ROH-based genomic inbreeding coefficients in JC goats. (**a**) The estimated effective population size of JC goats during the past 1000 generations. (**b**) Summary of ROH-based genomic inbreeding coefficients (F_ROH_) in JC goats. The plots (**c**–**f**) show the Pearson’s correlation between F_ROH_ and the partial inbreeding levels based on the ROHs with four different length categories (i.e., F_ROH 0.1–0.2 Mb_, F_ROH 0.2–0.5 Mb_, F_ROH 0.5–1 Mb_, and F_ROH > 1 Mb_) in JC goats.

Figure 3a shows that the estimated N_e_ exhibited a large decline from 5086 at 999 generations ago to 139 at 13 generations ago in JC goats. The total ROH-based inbreeding coefficients (F_ROH_) per animal ranged from 0.134 to 0.264 in JC goats, with an average value of 0.178 (Figure 3b). Figure 3b shows that the total inbreeding levels mainly resulted from inbreeding at 250–500 generations (F_ROH 0.1–0.2 Mb_) and 100–250 (F_ROH 0.2–0.5 Mb_) generations ago at the population level. Although there was a significant correlation between F_ROH_ and each partial F_ROH_ (i.e., F_ROH 0.1–0.2 Mb_, F_ROH 0.2–0.5 Mb_, F_ROH 0.5–1 Mb_, and F_ROH > 1 Mb_), the correlation analyses (Figure 3c–f) revealed that the variations in F_ROH_ across individuals were largely due to the differences in inbreeding that occurred 50–100 generations (*r* = 0.96, *p* = 2.2 × 10^−16^) and 50 generations (*r* = 0.91, *p* = 2.4 × 10^−12^) ago. Furthermore, F_HOM_ varied from 0.189 to 0.386 in JC goats, which showed a significantly positive correlation (*r* = 0.60, *p* = 4.1 × 10^−4^) with F_ROH_ (Supplementary Materials Figure S4).

### 3.5. Detection of ROH Islands and Selection Signals in JC Goats

**Figure 4:**
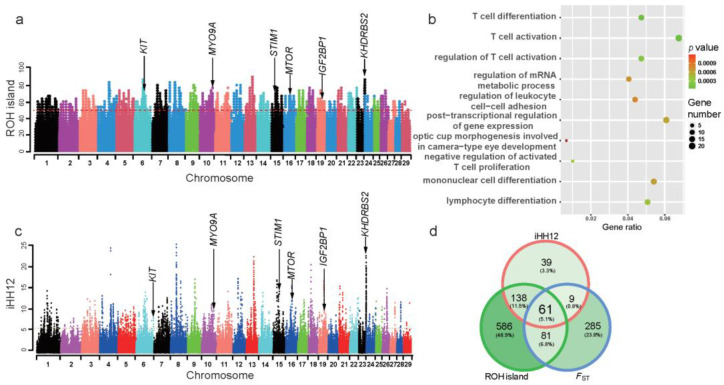
Genome-wide detection of ROH islands and selection signals in JC goats. (**a**) Manhattan plot of the genome-wide frequency of SNPs occurrence into ROHs in JC goats. The red line indicates that the SNPs in ROHs were shared by more than 50% of the animals, which was used to define ROH islands. (**b**) The top 10 enriched GO biological processes for the genes overlapping with ROH islands. (**c**) Manhattan plot of genome-wide iHH12 across all 29 autosomes in JC goats. The dashed line represents a threshold of the top 0.5% of outlier windows. (**d**) Venn diagram for the positively selected genes identified via three approaches.

ROH regions shared by more than 50% of the animals (i.e., the top 1.68% of the SNPs observed in all ROH) were defined as ROH islands (Figure 4a). Therefore, we detected 501 ROH islands with a minimum length of 1496 bp on 29 chromosomes that overlapped with 866 annotated genes (i.e., Ensembl ID) (Supplementary Materials Table S6). The two most common ROH islands (shared by 28 of 30 samples) were all located on Chromosome 13 (49,835,817–49,848,520 bp and 49,867,543–49,917,358 bp), but these regions were annotated without any known genes. The longest ROH islands with 1.04 Mb was observed at 47,562,462–48,604,174 bp on Chromosome 23 and harbored two genes (i.e., *KHDRBS2* and *RF00026*). The annotated genes overlapping with the total ROH islands were significantly enriched (*p* < 0.05) in 467 GO biological processes (Supplementary Materials Table S7). In the top 10 enriched GO biological processes, seven biological processes were immune related, such as T cell activation (20 genes, e.g., *IL2*, *IL7*, *KIT* and *TP53*), T cell differentiation (14 genes, e.g., *IL7*, *MTOR*, and *TP53*), and regulation of T cell activation (Figure 4b). Additionally, the *IGF2BP1* gene overlapping with an ROH island on Chromosome 19 deserved more attention because of its versatile biological functions.

**Figure 5:**
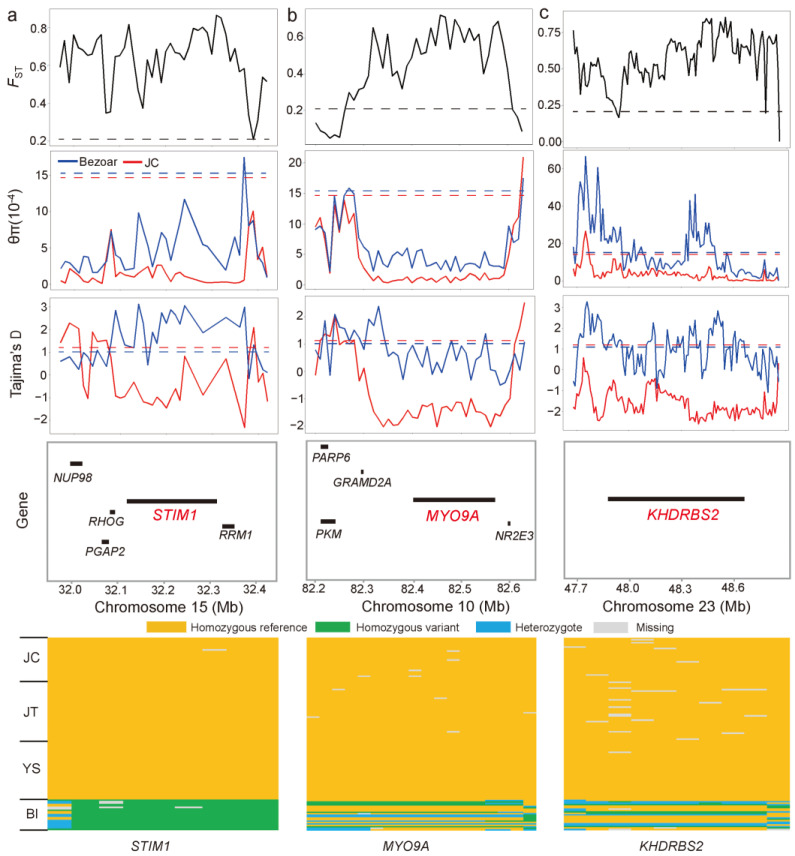
Summary of three examples of selective sweep regions. Three selection signals encompass *STIM1* on Chromosome 15 (**a**), *MYO9A* on Chromosome 10 (**b**), and *KHDRBS2* on Chromosome 23 (**c**), respectively. The putative sweep regions were additionally validated by π and Tajima’s D values. Horizontal dashed lines represent the whole-genome mean for the corresponding parameters. Gene annotations in the sweep region and SNPs fixed for reference alleles in three black goat breeds are indicated at the bottom.

To accurately search for positive selection signatures in JC goats, we applied iHH12 scores and *F*_ST_ (JC vs. Bezoars) with a threshold of top 0.5% of windows (i.e., iHH12 > 7.10 and *F*_ST_ > 0.68) (Figure 4c and Supplementary Materials Figure S5), in addition to ROH islands. Accordingly, we identified a total of 61 positively selected genes (PSGs, Supplementary Materials Table S8), which were significantly enriched in 384 GO biological processes (*p* < 0.05). Many GO terms were immune related, such as the regulation of alpha-beta T cell proliferation (2 PSGs, *CD274* and *JAK2*), alpha-beta T cell activation (3 PSGs, *MTOR*, *CD274*, and *JAK2*), and immune system development (6 PSGs, e.g., *MTOR*, *CD274*, and *KIT*) (Supplementary Materials Table S9). According to their biological functions and the distributions of SNPs within the corresponding regions across the sampled goat populations, several genes may be relevant for important traits in JC goats. For example, *F*_ST_ was very high (average windowed *F*_ST_ = 0.74) in the genomic region encompassing *STIM1* at 32,118,087–32,314,109 bp on Chromosome 15, which was supported by lower values of π (average π = 1.40 × 10^−4^) and Tajima’s D (average value = −0.84) (Figure 5a). There were 333 biallelic SNPs within the *STIM1* gene, among which 184 SNPs, including one missense substitution (c.5G > C, p.Arg2Pro; chr15:32,230,281) in exon 1, were fixed for the reference allele in JC goats. Strikingly, eight SNPs were fixed for the reference alleles in all 111 domestic goat samples, while the mutant alleles at these SNPs reached fixation in Bezoars (Figure 5a and Supplementary Materials Table S10). As shown in Figure 5b, the *MYO9A* gene (Chromosome 10: 82,392,953–82,577,244 bp) was under strong selection (average windowed *F*_ST_ = 0.71), which also showed lower π and Tajima’s D values. Overall, 18 SNPs in *MYO9A* are fixed for the reference alleles in the three black breeds (Figure 5b and Supplementary Materials Table S10). In addition, *KHDRBS2* (Chromosome 23: 47,871,981–48,673,939 bp) fell in a clear selective sweep that contained some windows with extremely low Tajima’s D values (average value = −1.62) (Figure 5c). The reference alleles at 10 SNP sites within *KHDRBS2* reach fixation in all three black breeds (Figure 5c and Supplementary Materials Table S10).

## 4. Discussion

In this study, we used high-density SNPs obtained from WGS data to investigate the genetic relationships of JC goats with two other Chinese black breeds (i.e., JT and YS) and Bezoars. The JC samples included in this study represent a substantial part of the paternal genetic material in JC goats from a core breeding farm. According to population structure and genetic differentiation analyses, JC goats were genetically distinct from two other Chinese black breeds and Bezoars, despite the geographical proximity. Among diverse evolutionary forces (e.g., artificial selection, adaptation to specific environments, and genetic drift), geographical isolation is the primary factor shaping genetic partitioning in worldwide domestic goat populations [2]. In Southwest China, the mountains and hills create natural barriers and restrict the spread or trade of goats in the Liangshan Yi Autonomous Prefecture and adjacent regions, which geographically isolate the JC goats and contribute to the unique pattern of genomic variations in this breed. Furthermore, JT and YS goats showed a very close genetic relationship and a substantial gene flow, in line with the active trade of breeding stocks between the two breeds.

Here, we characterized the genome-wide ROH pattern in JC goats by mining information from WGS data. The number of ROHs in JC goats is much more than those in many other goat populations [13,39,40,41]. Although population-specific genetic composition underlies this difference, another important reason is the setting of the minimum length of ROH, which varied with the different densities of SNP maps (i.e., SNP chips vs. WGS). Besides goats [13,39,40,41], previous studies using SNP chip data usually detect long ROHs (e.g., > 0.5 Mb [12,42,43] or 1 Mb [11,44,45,46]) in a variety of livestock populations. In contrast, the WGS data usually yield millions of SNPs and enable us to detect short ROH with a length of < 500 kb, as demonstrated in cattle [47], goats [14], and sheep [32]. Our LD decay analysis implied that ROHs with a minimum length of 100 kb substantially excluded the ROH artifacts that may result from high LD between SNPs [31]. Strikingly, the proportion of ROHs with < 200 kb in size was very high, and the distribution of ROH length approximated an L-shaped distribution in the genomes of JC goats, consistent with the results in global goat populations [39] and several Chinese goat breeds [40]. We also observed that the highest number of ROHs per chromosome was on Chromosome 1, in line with the findings in goats [40], cattle [48,49], sheep [12], and pigs [45]. The number of ROHs on each chromosome showed a strong positive correlation with the chromosome length in JC goats, suggesting that the distribution of ROH was uniform across chromosomes. Although inbreeding, selection, and other evolutionary factors can drive the formation of ROHs, individual or combined effects of evolutionary factors would lead to the different distributions of ROHs over the genome. For example, the distribution of ROHs across chromosomes is more variable in populations under intensively artificial selection than those in unselected cattle [50,51]. Furthermore, dense ROH-peak regions among the cattle result from the collective effects of inbreeding and selection [47]. Given the breeding history of JC goats, the genome-wide uniform distribution of ROHs is mainly due to inevitable inbreeding in the relatively small population size of this breed.

In the current work, we also examined the inbreeding levels in JC goats principally using F_ROH_. One advantage of genomic inbreeding estimates (e.g., F_ROH_) over pedigree-based coefficients is that they can capture inbreeding produced by distant ancestors not included in the pedigree [52,53]. A large-scale analysis using SNP chip data [39] reported that ~ 60% of 117 goat populations showed low inbreeding levels (F_ROH_ < 0.1), whereas ~30% of the breeds showed moderate F_ROH_ values (0.1 < F_ROH_ < 0.2). Accordingly, all JC goats showed moderate (0.1 < F_ROH_ < 0.2) or high F_ROH_ coefficients (F_ROH_ > 0.2), similar to the results in 11 Swiss goat populations [14]. Notably, high F_ROH_ values could be partly ascribed to abundant short ROHs (e.g., < 0.5 Mb) identified in JC goats and Swiss breeds using dense SNPs obtained from WGS. If we excluded the fraction of short ROHs, the total F_ROH_ values were generally low and comparable to the findings in several Chinese breeds [40]. Particularly, the variations in the inbreeding coefficients across JC goats mainly result from inbreeding events during the past 100 generations, also supported by the relatively small N_e_ in these generations. Moreover, the historical trend in N_e_ of JC goats agreed with those in other Chinese native goat breeds [40,54], mainly attributed to a lack of high commercial values of these breeds and changes in the economy in China. Consistent with previous studies in livestock [15,53], we also found a high correlation between F_ROH_ and F_HOM_ because both measures capture whole-genome homozygosity irrespective of the allele frequency at different genomic positions.

As a merging index, ROH islands are increasingly used to search for selection footprints in livestock species. Infectious diseases frequently affect livestock health and production performance during the whole life span, which implies that natural selection likely acts on immune-related genes. Here, many immune-related genes (e.g., *IL2*, *IL7*, and *TP53*) overlapped with ROH islands in JC goats, in line with the selection mapping analyses in other goat breeds [55,56]. We also found that one ROH island in JC goats encompasses *IGF2BP1* encoding an RNA-binding protein, which was supported by high iHH12 values. *IGF2BP1* plays an important function in animals via regulating cell proliferation, differentiation, and morphology and thereby contributes to intestinal health and body weight in mice [57]. Large-scale genomic studies demonstrated that regulatory mutations upstream of *IGF2BP1* are plausible causal variants that affect body size in chickens [58] and ducks [59].

We further integrated three complementary outlier approaches to search for the putative signatures of selection in JC goats. Among the selection signals detected in JC goats, a selective sweep harboring *STIM1* deserves more attention. Previous studies demonstrated that *STIM1* is under strong selection in many goat populations worldwide [14,16,56]. Our detailed inspection revealed that eight biallelic SNPs within *STIM1* are fixed for the reference allele in three black domestic breeds, while the Bezoars only carry the mutant alleles. Interestingly, the *STIM1-RRM1* haplotype in domestic goats was putatively introgressed from an ibex-like species and linked to neural function or behavior [56]. However, the target traits associated with *STIM1* are still not clear in goats and need to be explored in future work.

Detection of the genetic loci affecting reproduction traits (e.g., litter size) in goats has long been of interest. Here, we identified several selected genes related to reproduction, such as *KIT* and *KHDRBS2*. The *KIT* and its ligand (i.e., *KITLG*), two well-known pigmentation genes in livestock, are common selection signals in many different goat [16,18,54,60,61] and sheep populations [18,62], which may imply their pleiotropic effects. We previously identified a selection signal encompassing *KHDRBS2* in Meigu goats [63], which are known for their high fertility and precocious puberty. In Chinese Arbas Cashmere goats, one SNP in *KHDRBS2* shows a genome-wide association with litter size [64]. Previous GWAS studies reported that the SNPs and indels within or near *KHDRBS2* were significantly associated with reproduction traits in cattle [65] and pigs [66]. In summary, increasing lines of evidence support the supposition that *KHDRBS2* is likely to be a candidate gene affecting reproduction traits in livestock.

## 5. Conclusions

Among the three black goat breeds included here, JT and YS showed a close genetic relationship with a non-negligible amount of gene flows but they were genetically distant from JC. The historical effective population size of JC goats continuously decreased during the past 1000 generations, particularly in the last 100 generations. According to ROH-based inbreeding values, JC goats generally showed moderate inbreeding levels. This study identified several putatively selected genes in JC goats, which may underly genetic adaptations to local environmental conditions and may be associated with important traits such as immune response, behavior, and reproduction.

## Data Availability

The data presented in this study are available on request from the corresponding authors.

## Supplementary Materials

The following supporting information can be downloaded at: https://www.mdpi.com/article/10.3390/ani12182365/s1, Figure S1: A line chart of cross validation errors in the admixture analysis of 132 sampled goats; Figure S2: Box plot of genome-wide *F*_ST_ in 10-kb non-overlapping windows between JC and two other goat breeds and Bezoars; Figure S3: Genome-wide LD decay between SNPs in JC goats; Figure S4: The summary of F_HOM_ and its correlation with F_ROH_ in JC goats; Figure S5: Manhattan plot of genome-wide *F*_ST_ in 10-kb non-overlapping windows between JC and Bezoars; Table S1: Summary of the short-read sequencing data and mapping statistics for three black Chinese goat breeds and 21 Bezoars; Table S2: Distributions of the identified biallelic SNPs in 132 goat genomes within various genomic regions; Table S3: A genomic relationship matrix in JC goats; Table S4: The summary of the total ROH number and the total ROH length in the genomes of 132 goats; Table S5: Summary of the linear relationship between the chromosome length and the total ROH length or the total ROH number per chromosome in two other black breeds and Bezoars; Table S6: Summary of ROH islands and the overlapping annotated genes in JC goats; Table S7: The significantly enriched GO biological processes for the genes overlapping with ROH islands; Table S8: Summary of the selected genes identified via three approaches; Table S9: The significantly enriched GO biological processes for the selected genes; Table S10: Summary of SNPs within three selected genes fixed for reference alleles in three black goat populations.
