# Supplementary material for: Genetic Diversity and Selection Signatures in Jianchang Black Goats Revealed by Whole-Genome Sequencing Data
Source: Animals (Basel). 2022 Sep 10;12(18):2365. doi: 10.3390/ani12182365 (PMC9495118; doi:10.3390/ani12182365)
Supplement: Supplementary file 1 [file animals-12-02365-s001.zip › Table S5.pdf]

Table S5 Summary of the linear relationship between the chromosome length and the total ROH length or the total ROH number per chromosome in two other black breeds and Bezoars

Breed	Relationship between the chromosome length and the total ROH length	Relationship between the chromosome length and the total ROH number
JT	b = 1.41 (R^2 =0.44, $P < 2.2\times10^{-16}$)	b = 0.53 (R^2 =0.67, $P < 2.2\times10^{-16}$)
YS	b = 1.15 (R^2 =0.14, $P < 2.2\times10^{-16}$)	b = 0.44 (R^2 =0.28, $P < 2.2\times10^{-16}$)
Bezoars	b = 1.62 (R^2 =0.13, $P < 2.2\times10^{-16}$)	b = 0.46 (R^2 =0.29, $P < 2.2\times10^{-16}$)

Note: The unit of the total ROH length and the chromosome length were 100 Kb and Mb, respectively.
