# Supplementary figures and images for: Genetic Diversity and Selection Signatures in Jianchang Black Goats Revealed by Whole-Genome Sequencing Data
Source: Animals (Basel). 2022 Sep 10;12(18):2365. doi: 10.3390/ani12182365 (PMC9495118; doi:10.3390/ani12182365)

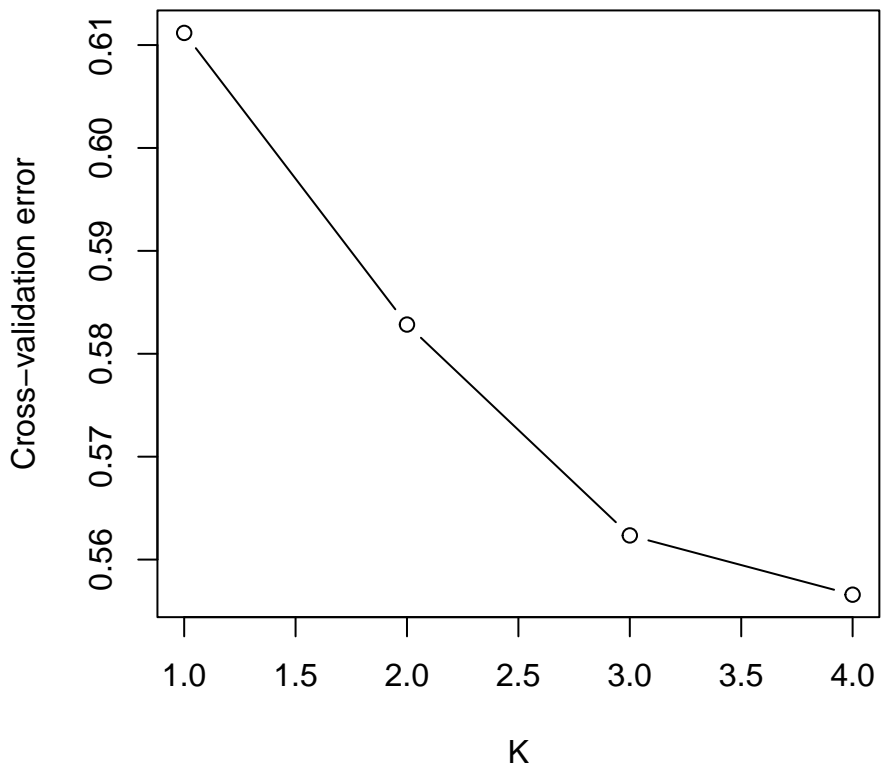

Supplement: Supplementary file 1 [file animals-12-02365-s001.zip › Figure S1.pdf]

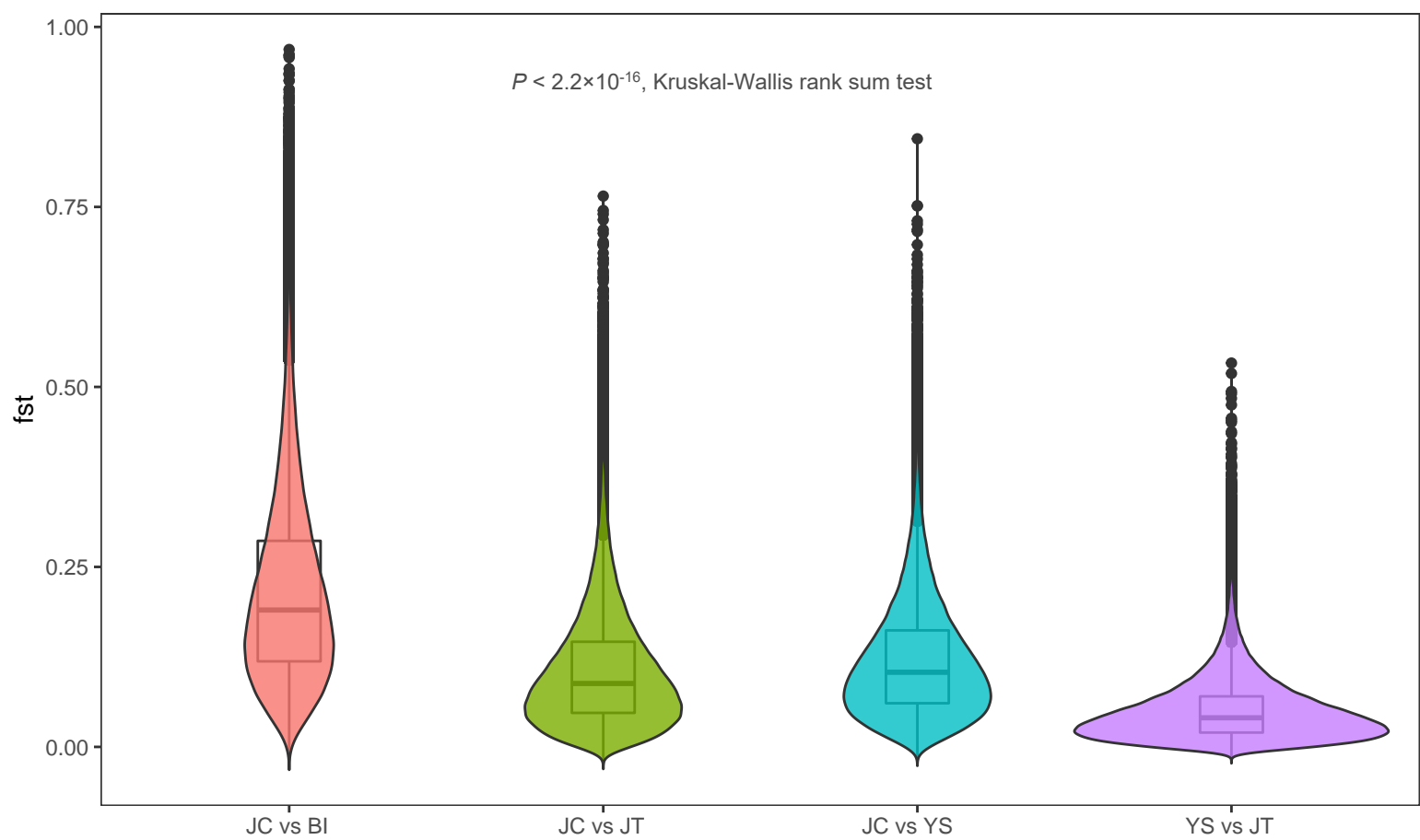

Supplement: Supplementary file 1 [file animals-12-02365-s001.zip › Figure S2.pdf]

## LD decay

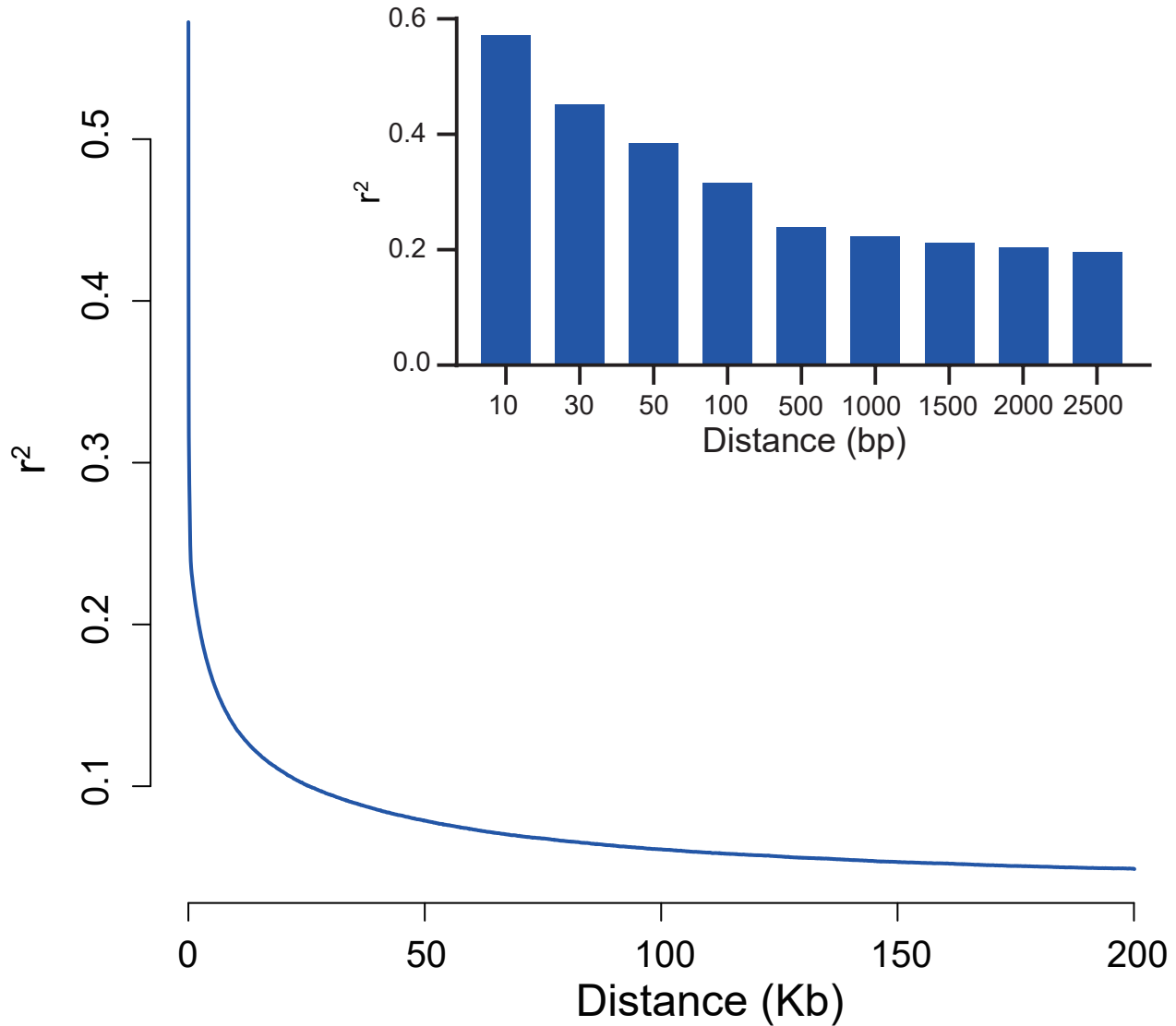

Supplement: Supplementary file 1 [file animals-12-02365-s001.zip › Figure S3.pdf]

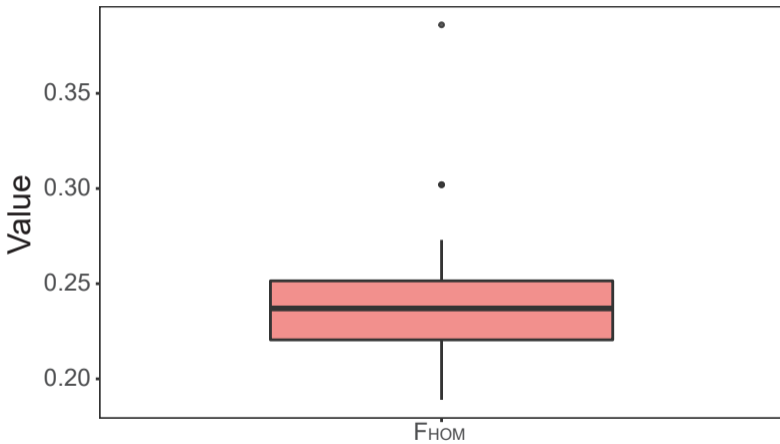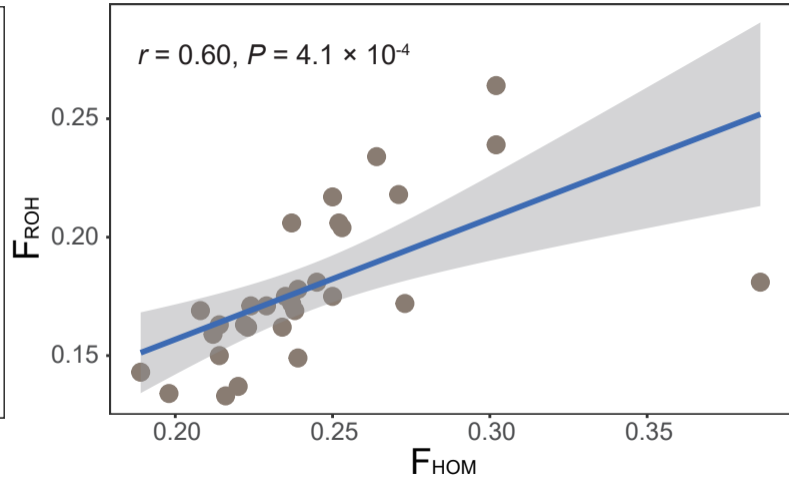

Supplement: Supplementary file 1 [file animals-12-02365-s001.zip › Figure S4.pdf]

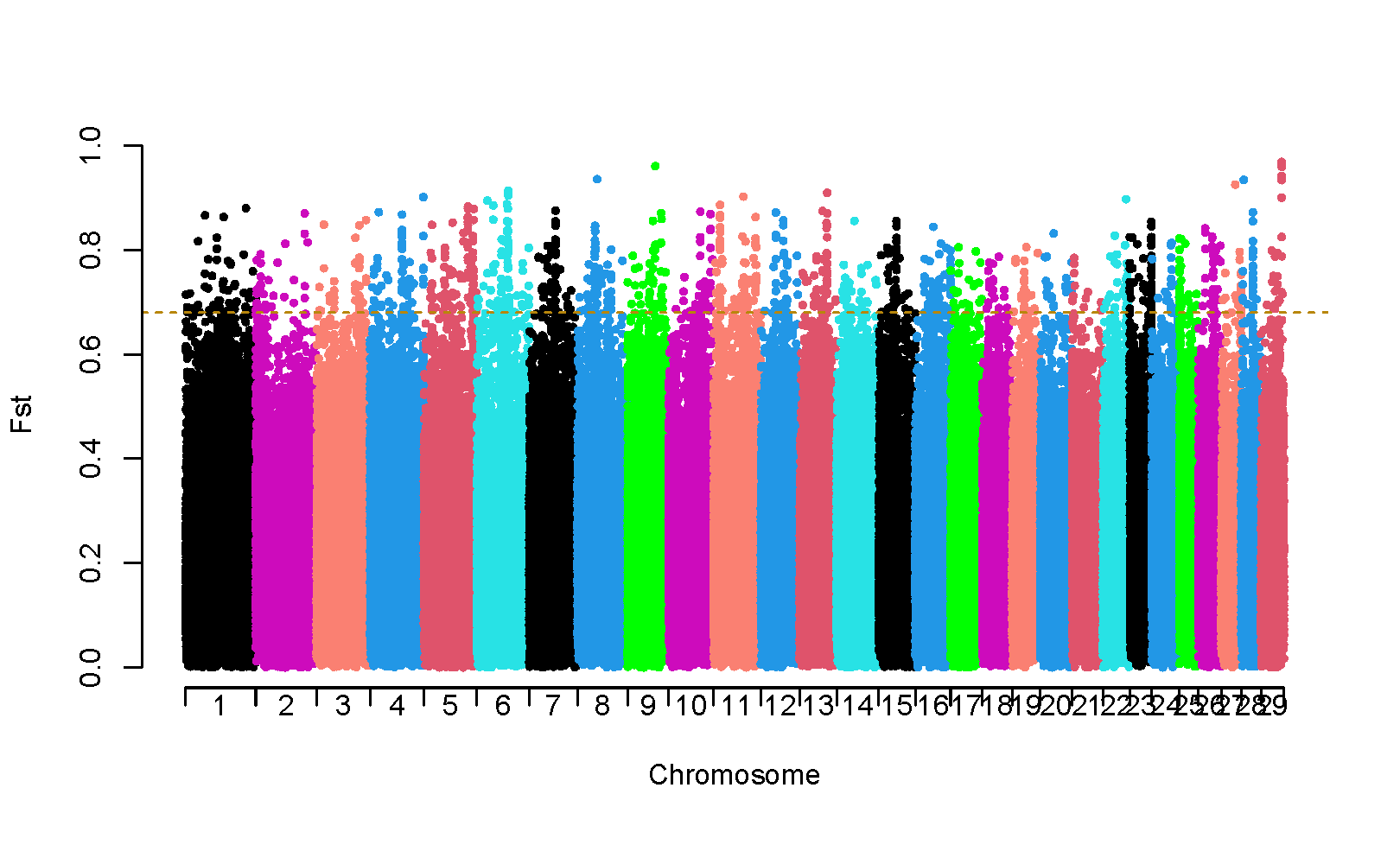

Supplement: Supplementary file 1 [file animals-12-02365-s001.zip › Figure S5.tiff]
